# Quarantine an Advertiser

**Published:** 1879-09

**Authors:** 


### QUARANTINE AN ADVERTISER.

We suggested not long ago that it would be very poor argument for the future prosperity of Little Rock, to assume that it is as liable to the yellow fever pestilence as Memphis, and that our only protection is quarantine; but that it would be a very strong argument in our favor to present the real facts, that we are protected by geographical position and other natural causes. It is pleasant to see so good a reasoner and honest journalist as the editor of the Arkansas *Democrat*, express in the following language a similar sentiment:

"While we rejoice in our exemption from the scourge, it should not escape us that the means employed for our safety is a public advertisement to the outside world of the danger that surrounds us. It is a confession of our weakness, and to neglect these things (sanitary cleansing) and depend on quarantine for safety, year ater year, will simply end in ruin to our highest interests."

It would be difficult to state a point of fact in more pointed and logical terms than this. And the ruin is being demonstrated every day. Stagnation of business, the people scared to death and ready to run—made to believe by sensationists who never read a doctor book, that we are on a volcanic mine of pestilence, only waiting to be fired by some Memphis refugee, and that such refugees are more poisonous than the deadly upas leaf. Only two weeks ago the result of this unwarrantable panic was the death of a young lady of Little Rock, who left town on the Fort Smith train, got off at Sparta, failed to meet an expected brother, and walked in the heat to a neighboring house, had used too much ice water and was overheated. The people of the house were alarmed and left her alone, to die of overheat and exhaustion. Who is responsible? Not the doctors. Quarantine as we have it is instigated by sensationists. Doctors, if let alone, would quarantine against fabrics, vessels that carry bilge water, and other things known to be vehicles of portability, and use sanitary means to prevent the air from being so impure as to be impregnated with the poison, should it be conveyed by such means, and not against persons.

Two weeks ago the *Scientific American* said: "There is probably no man living whose competency to discuss the subject of yellow fever is more widely recognized than Professor Alfred Stille, of the Philadelphia medical school. In his last great lecture he says: 'It is not contagious, but portable in vessels and fabrics, not in the human system;' that the infection can only spread when climate and surroundings are favorable. He quotes Dr. Nott, and says: 'The late Dr. Nott, who spent nearly all of his professional life in Mobile, and whose competency in such a question no one will doubt, states his judgment thus: 'Yellow fever is not generated in the human system, *nor transmitted from one person to another, in any way;* its germ or poison is generated outside of the human system, and is taken into the system after the manner of the marsh malaria poison. But unlike the latter, the germ is portable, and may be carried from one point to another, and thus propagated.' And again, he says: 'Few of the old and experienced physicians of the yellow fever zone believe in the contagiousness of the disease, and their convictions are based upon facts coming under their observation. During thirty years' residence in Mobile my experience corresponds with theirs.'

"The late Dr. Warren Stone, of New Orleans, who probably had more experience of yellow fever than any man who ever lived, stated emphatically the exact truth when he declared: 'I am perfectly convinced beyond a doubt or hesitation, that, personally, it is not contagious; I *know* that it is not.'"

But says Professor Stille, and all others of medical attainments, it is infectious and portable, and strict quarantine will prevent its dissemination. What does that mean? It means that the poison taken from an infected port, in vessels or fabrics, to another place where the atmosphere is liable by temperature, filth and otherwise to be infected and inhaled, then the disease will spread.

By strict quarantine the doctors mean, keep vessels and fabrics of infected ports that carry the poison away from places where temperature, humidity of air and filth and low altitude would render the epidemic likely, but fumigate and purify your traveler, give him a new suit of clothes, and let him pass on. That is science, common sense and policy, and a mixture of benevolence to the extent of a suit of clothes. No running of blockades then. As it is, no mortal man can feel another's pulse, and look at his tongue, at St. Louis or Chicago, and tell whether he comes from Memphis or New York, unless he wants to tell it himself. The same of boxes of merchandise. But Professor Stille says, "It is difficult to get us to distinguish between a ship and its crew, and between people and their clothing." And yet there is a very distinctive difference. The ship or clothes convey the fever germ, the crew or people are afflicted by its poison. The same difference as that between a soldier wounded and the minnie ball that struck him. There is danger on the field of fight, but none in dressing the wound a hundred miles away. Danger of billious or intermittent fever if you stay ten miles below Little Rock, but none in waiting on the patient if he comes to town. The same of yellow fever, if the distinguished medical men of the world can be relied on.

Are we then in favor of quarantine for Little Rock? Yes, of the character stated—upon the principle that prudence is the better part of valor, and our sanitary condition and geographical position may not justify us in taking risks. But a quarantine of a character admitted to be ineffective, and every few days unavoidably violated, only proves that it is not the protection, but that we have natural defenses that are effective when other efforts fail. This has been demonstrated here for the past fifty years.

If the newspapers and sensationists will do half as much to convince the world of this fact as they have done to try to prove that we are as bad off naturally as Memphis and only protected by quarantine, then merchants, manufacturers, and all sorts of business men, will make this the business and pleasure metropolis of the southwest, and in a few years we will have fifty thousand inhabitants, and the people will not be afraid to stay at home and invite others here as the place of safety, pleasure and prosperity.

Little Rock is not located for a fever town, and we do not believe it would prevail here as an epidemic if we had fifty thousand inhabitants, and cases were to die here, as they have in the past, who contracted the disease elsewhere, provided sanitation is not neglected in the mania for quarantine.

We are thus earnest solely for the vindication of the truth of medical science, and for the good of this country and the cause of humanity. We would not pull down a laurel from the brow of the faithful quarantine advocate, for his purposes are good, even if he is mistaken in his remedy. But the whole country is suffering from its effects, and if continued "from year to year," will end in stagnation and ruin. Our population will grow smaller instead of larger, for we cannot stand the pressure of evils that follow in the train of blockade and exciting fears of impending woes.—*Sunday Visitor.*

